# Going beyond: an exploration of residents’ experiences in recovery and homelessness supported housing provision in the United Kingdom

**DOI:** 10.3389/fpubh.2026.1783501

**Published:** 2026-04-20

**Authors:** Amelia Simpson, Hannah Maiden, Reuben Larbi, Sandra Bee, Caitlain Biricz, Andrew Harding

**Affiliations:** 1 Division of Health Research, Lancaster University, Lancaster, United Kingdom; 2 Health Determinants Research Collaboration, Blackpool Council, Blackpool, United Kingdom; 3 Healthwatch Blackpool, Empowerment Charity, Blackpool, United Kingdom; 4 BoingBoing Foundation, Blackpool, United Kingdom

**Keywords:** homelessness, housing, mental health, recovery, supported housing

## Abstract

Appropriate support is essential for the recovery of individuals with complex needs. In the United Kingdom, individuals with multiple complex needs such as those experiencing homelessness or recovering from substance use reside in supported housing. This type of housing is provided by a range of housing associations, local authorities, and charities. Often, a proportion of these individuals require tailored support to address their overlapping needs relating to health and daily living. However, there is a paucity of United Kingdom research on this topic. The aim of this present study is to explore the experiences of residents’ living in recovery and homelessness supported housing provision. Semi-structured interviews were conducted with 11 residents from a coastal town in the North-West of England. Data collection occurred between February and March 2024. To analyze the interviews, thematic analysis was used. Two overarching themes were identified. The first was “going beyond” which unpacked into four subthemes “compassionate care,” “availability of support,” “comfort and safety,” and “personalized approach.” The second theme was “community and relationships” which unpacked into three subthemes “other residents,” “activities” and “stigma.” Findings showed that tenants valued staff who provided support beyond their formal roles, especially those with lived experience. The findings also highlight the importance of housing conditions, personal autonomy, and community engagement in fostering a sense of home and identity. The implications of this study highlight the need for service models that extend beyond basic care.

## Introduction

Housing is a key determinant of health and well-being (1, 2). Yet many groups encounter housing instability and need to access and engage with supported housing. The United Kingdom government defines supported housing as accommodation that is provided along with support, supervision, or care to help people live as independently as possible. Supported housing exists to support people often with multiple and overlapping needs, including older people who can no longer live at home (3–5); have a diagnosis of dementia (6); people with mental health issues (7, 8); those who have experience of the criminal justice system; individuals and families at risk of homelessness; and domestic abuse; and those in recovery from drug and alcohol dependence (9). In the United Kingdom, supported housing is a mixed economy of provision, mostly provided by housing associations and local authorities, but also charities, the voluntary and private sectors (10). The United Kingdom National Audit Office (11) estimates 651,000 supported housing units in the United Kingdom, of which 85% are in England (based on a 2016 government review) and has associated costs of £3.5 billion per year.

Given that supported housing serves many groups, often with multiple complex needs, unsurprisingly existing literature is disparate. Complex needs can be defined as any adult who requires a high level of support across multiple aspects of their daily life and who rely on a range of health and social care services (11). However, for supported housing research in the context of homelessness there is an international evidence base, although there are also notable gaps. The international evidence base highlights how supported housing provides a foundational place of safety where people can, with the right support, begin to rebuild their lives. A high proportion of the evidence base that demonstrates mixed efficacy is from North America and mostly focuses on provision for military veterans and schemes delivered through the federal Collaborative Initiative on Chronic Homelessness (12). While limited to the Norwegian context, Nesse et al. (13) highlight the role staff play in recovery and life satisfaction. However, it is noted a systematic review that the United Kingdom is poorly represented in the international evidence base on supported housing concerning groups who are homeless and or can be in recovery (12).

A 2018 systematic review included no research from the United Kingdom in respect of supported housing for homeless people (12). A more recent systematic review published in 2024 on recovery experiences of homeless people with substance use disorder found 15 papers, only three of which report research undertaken in the United Kingdom (including one with a partial focus on the United Kingdom) and only two of these were published since 2015 (14). Another relevant systematic review published in 2024 of service users’ and providers’ experiences and perceptions of mental health accommodation services found 43 papers, with only one reporting United Kingdom research (15). This limited United Kingdom evidence focuses on conceptualizations and meanings of personal recovery (14), residents’ social capital (16, 17) and a strength-based approach for those encountering homelessness (18). With limited evidence, United Kingdom researchers have developed a logic model that highlights the hypothesized impact housing interventions have for homeless people, including intermediate outcomes, moderating factors and more distal outcomes (12). However, ultimately the logic model is based on moderate to low-quality evidence mostly from outside of the United Kingdom and on housing interventions which are inclusive of supported housing but not specific to it (12, 14, 15).

With complex provision, and with the nature of overlapping and multiple needs of residents, it is difficult to contextualize, frame, and interpret evidence in respect of national context and population group. It is noted that governance is an underrepresented dimension in supported housing research (12). Subsequently, it is important to situate research in the relevant policy and governance context. Broader United Kingdom state intervention in housing predates the welfare state by at least 50 years (19), and yet commentators have referred to housing as the ‘wobbly pillar’ of the welfare state because it is seen as being on the periphery of welfare policy (20). Specifically on homelessness, direct state intervention was established long after welfare intervention in other areas such as education, health, and social security. Currently, policy and the governance structures that overlie supported housing for homeless people can be considered multiple, complex, and reliant on the priorities of several central and local government actors (19). For example, while the Department of Health & Social Care develops policies that aim to enable independent living, it is The Ministry of Housing, Communities and Local Government (formerly The Department for Levelling Up, Housing & Communities) who are responsible for the supply and quality of supported housing (21). In addition, The Department for Work & Pensions (DWP) reimburses local authorities (who manage provision in their area) for paying housing benefit claims. Within the context of complexity with respect to the needs of the population group and the overarching policy context, this research aims to explore residents’ experiences of recovery and in homelessness supported housing provision in the United Kingdom.

## Methods

### Context

The study took place in the North-West of England at recovery and homelessness supported housing provision. The supported housing provision in this study is in a coastal town with extremely high levels of social and economic deprivation, some of the poorest health outcomes in the country, a poor housing stock, and a relatively transient population. Given this location is a small town, and while it is important to contextualize this research, we do not provide references or demographics to protect the anonymity of participants.

### Participants

Residents who lived in either recovery or homelessness supported housing provision from across five sites were invited to participate. Staff shared the poster invitation and participant information sheet with potential participants. Eleven participants were recruited using purposive sampling. Participants were given a £10 gift voucher as compensation for their time.

### Data collection

A qualitative approach using semi-structured interviews was used to explore participants’ experiences of living in supported housing following ethical approval from Lancaster University Faculty of Health and Medicine Ethics Committee. Interviews were conducted between February and March 2024 at a time agreed upon by the participant. After gaining verbal consent, the interviews were conducted by two researchers. The first author led the interview and asked the questions, while the second interviewer asked follow-up questions where appropriate. Interviews lasted 25 min to an hour, were audio recorded, and transcribed verbatim.

### Analysis

The recordings of the interviews were transcribed, and transcriptions were securely electronically stored. The data was analyzed following the six steps of reflexive thematic analysis approach (22). Data was analyzed inductively, meaning it was not shaped by pre-existing theory. Initially, the first author familiarized herself with the data and coded according to the interview guide topics. Following this, codes were then analyzed into provisional themes when they had shared similarities. The first author then met with the rest of the research team to discuss and reflect on the initial themes and subthemes. The research team engaged in critical reflection to finalize the themes.

### Ethical considerations

Written consent was required for participation in the interviews. Before the recording started, participants were informed about their right to withdraw without a reason or consequence and that their participation was voluntary. Staff were not present during the interviews and verbal consent was taken from the participants prior to the interview starting. All data was anonymized before sharing with the wider research team.

## Results

The following section outlines the two themes and seven subthemes that resulted from the thematic analysis. The direct quotes from the interviews are displayed using pseudonyms. These themes are (i) going beyond and (ii) community and relationships.

### Going beyond

This overarching theme captures the multifaceted nature of support provision, contributing to and affecting a resident’s experience. It reflects how staff exceed care expectations and go the extra mile for residents. There are four subthemes within this theme: (i) compassionate care, (ii) availability of support, (iii) comfort and safety, and (iv) personalized approach.

#### Compassionate care

This subtheme emphasizes how staff display empathy and understanding towards residents. Residents sometimes expressed frustration with staff interactions lacking empathy. It suggested staff may benefit from a deeper understanding and patience when residents exhibit challenging behaviors.

*“We’re not doing it because we think it’s a smart thing to do, but because we are just kids at the end of the day. We’ve had bad, we have had shit childhoods, so of course we are gonna act up…We’re doing it because that’s what we think, what we have been brought up to think is right.”* (Carl)

Residents emphasized how staff went above and beyond to monitor and notice changes in behavior among residents. It highlights a proactive approach taken by staff to check in and provide support if a resident was acting out of character. *“So then [staff] and [staff] will come in, they always ask how’s such-a-body, have you seen such-a-body, and we’ll say we have not seen them for a couple of days, and then they go and either ring them or knock on the door and see if they are alright”* (Lisa).

#### Availability of support

This subtheme recognises the benefit of reliable and available support. Residents emphasized the importance of having access to round-the-clock support. They expressed an appreciation for the dedication of the staff, highlighting how they made themselves available to residents when they needed it. This accessibility provided reassurance to the residents knowing they could reach out at any time. *“If you are feeling down…they’ll just sit there and talk to you, or if they are not available you can send them a text and they’ll get back to you”* (Daniel).

#### Comfort and safety

This subtheme describes how creating a sense of home and safety for residents was important. Residents appreciated the autonomy to customize their living space to their preference if they wanted. *“We’re allowed to customize our rooms if we get the permission and the money ourselves, so they are also willing to help you with that”* (Ava). Security equipment played an essential role in fostering a sense of safety for residents. *“I feel safe in my flat. There’s a camera directly at the front door outside, there’s one at the back door, there’s three, there’s four in the building”* (Felix). For residents in recovery housing, the housing space was seen as symbolic and represented transformation in their recovery journey. This suggests the importance of creating a nurturing environment that not only provided a roof over their head but represented their future. *“I was in the other housing, and when I was in there I’d look around and it was like still being in addiction, the houses were just not up to scratch, dirty carpets, do you know, dirty walls, half decorated. And then coming here was like wow”* (Lisa).

#### Personalized approach

This subtheme highlights the importance of staff understanding residents’ unique needs and preferences. One resident shared how staff had gone the extra mile to personalize their Christmas experience which had left a marked impact. Personalized touches contributed to the residents feeling valued and appreciated for who they are.

*“We got these like massive food hampers, they were literally just a massive B&Ms bag full of food…And it wasn’t like everyone got the same thing, it was like everyone got like sort of the same thing but different…I like my hoodies, so I got a hoodie, some got caps and stuff, It was like they really thought about it.”* (Carl)

Residents valued the presence of staff members who had lived experience of substance use, homelessness, or supported housing. The shared background created a mutual understanding and residents looked up to these staff members as role models for their own journeys having seen them successfully transition into society. *“Knowing, they’ve got wise words, they’ve been there before, that I keep coming back to that because that is massive. They understand, they’ve been there. There’s nothing that we can say to them that they think oh, i’ll be shocked by”* (Lisa).

### Community and relationships

This theme captures the importance of interpersonal relationships between residents within their supported housing environment and the community around them.

#### Other residents

This subtheme explores residents’ relationships with each other where they bond over shared experiences but also experience challenges and conflict. Residents shared their experiences of living with others within the accommodation. They highlighted how trust was an important value to have. *“You’ve got to trust people, you cannot just go through it not being able to trust anyone, cause when you live with X people that’s a lot of people around, you have got to trust sometimes”* (Felix). Residents highlighted how shared experience helped them bond and offer support to one another. These common experiences served as a foundation for building empathy among the residents, demonstrating strength in each other’s company. *“Everyone’s so tight knit, because we have all been through the same type of situation”* (Jasper). Residents also faced challenges with each other. The challenges came from differences in personalities, needs, and points of their journey. “I *always use my manners wherever I go. But some people in X they do not know what manners are…A lot of residents I’ve spoke to treat [accommodation] like a doss hole”* (Mike).

#### Activities

This subtheme explores the variety of activities available to residents inside and outside of the accommodation and the benefits these activities provided for their well-being and sense of community. Residents expressed their appreciation for the variety of activities offered to them. *“We also do meditation and stuff, and sometimes we do step work as well…or listen to a podcast”* (Ezra). The activities provided to residents offered immediate and long-term benefits including a sense of purpose, establishing routine, and freedom from boredom. The activities served more than just a pastime but fostered a sense of belonging and fulfilment in residents’ lives.

*“We’re on, they were on about going abroad this year, so I’ve just put in for my passport, which I’ve never had a passport in my whole life…So yeah, all this stuff, it’s getting me into the real world, do you know what I mean? And that’s what we do, and yeah. I feel part of society today, I do!”* (Lisa)

#### Stigma

This subtheme details the resident’s experience around internalised feelings of shame due to their living situation or other aspects of their background. Residents were aware of the differences between themselves and their peers, as well as the stigma associated with living in supported housing. *“Then going to college, you make friends but oh what do I say to them? That I live by myself, do I live with my parents, you know stuff like that”* (Ava). Residents were frustrated when assumptions were made by staff about their future. The frustration stemmed from the perception of being unfairly judged based on their background.

*“You are not always gonna have that amount of money sitting around. And it’s like well, you do not know that, I could move out and become really successful…you do not know where my future goes, you are making it out as if I’ve got to live off the cheapest, I’ve always got to live off my benefits”* (Carl).

## Discussion

Within the context of population, deprivation, and policy complexity, this study aimed to explore the experiences of residents in two types of supported housing provision. The sample consisted of 11 residents who resided in either homelessness or recovery supported housing. The collection of detailed descriptions in this analysis makes possible for a wide range of relevant discussion. However, we have chosen to highlight the findings most congruent to existing literature.

Within the themes presented in the findings, the residents describe the staff’s ability to go beyond their role, ranging from providing round-the-clock access to support, sharing lived experiences, and facilitating autonomy. Previous literature has pointed to the central role that support staff play in the lives of residents, emphasising how the quality of relationships between staff and residents can impact health outcomes (23, 24). This is similar to the residents in our study who recognized the dedication of staff and valued the seemingly small acts of genuine interest and support. This is shown in other literature, which emphasizes the fundamental importance for users to be seen, heard, and respected as normal human beings by professionals, an approach regarded to impact their improvement (25). According to residents in our study, staff having their own lived experience were viewed as role models for their own recovery. The benefits of having lived experience within the workforce for users have been cited in the mental health literature, including increased independence and strengthened social networks (26). However, it is worth acknowledging that the effectiveness of professional relationships can be shaped by the support available to staff, including ongoing supervision which is essential when managing the emotional demands of the role (27). Given that concepts such as therapeutic alliance and helping relationships are well documented as predictors of outcomes in psychotherapy literature (25, 28), the above findings should not be surprising. However, these findings highlight how lived experience can be embedded into professional practice in helping relationships (25, 26).

Historically, homelessness and recovery housing have been in poor physical condition and existing literature suggests factors such as open or closed spaces, noise levels, and light can impact psychological well-being (29, 30). Furthermore, the psychological conceptualization of home extends beyond being a material object representing something that can provide autonomy and control (1, 31). Having the opportunity to have resources such as personal storage, access to a television, and furniture helps residents in supported housing feel engaged and establish social networks in the community (32, 33). From the perspective of ontological security, the home serves as a secure base where people can develop social identity and improve mental health outcomes (34, 35). Furthermore, the development of agency and personal autonomy are essential goals of many supportive and therapeutic interventions (36). It is suggested that when behavior is autonomous it is congruent to a person’s authentic interests and can improve well-being (37). Supported housing can provide a foundation for identity renegotiation through regaining agency and autonomy (38). Autonomy can be experienced through making everyday decisions and accessing the accommodation independently (39). These ideas are supported by the findings from residents in this study who were given the autonomy to customize their living spaces or were in well-kept accommodation which residents valued. This reinforces housing services as important in creating a sense of home and security.

The residents of this study touched on aspects of forming community around them. Residents emphasized the role of their peers in the facility in providing support during their journey. These findings align with previous research that participating in relationships of support with others contributes towards belonging and integration in a community (33). Residents in our study engaged in support roles with one another through sharing mutual experiences. Engaging in support roles and being a source of kindness for other residents have been identified as vital elements to becoming embedded in a community (33, 40). Moreover, having access to activities such as gym facilities and cooking lessons impacted residents’ well-being and sense of community. Participating in wider activities beyond supported housing has been cited as necessary to become socially included in the wider community through providing residents with experiences of social connection and interaction. This further supports that housing provides a foundation for individuals who live with complex needs to engage with activities outside of the accommodation (41). Having the opportunity to engage in activities, such as applying for a passport or attending a cooking class meant residents took on new responsibilities and contributed to them feeling a part of society. This supports how when satisfying one’s basic needs of shelter and safety, people can rebuild and focus on meaningful activities, suggesting the provision of such activities in supported housing can facilitate a turning point in meaning and identity (8, 42).

### Strengths, limitations and future research

One strength of this study is its social and geographic context. The accounts are contextual to the housing challenges of a town with significant social and economic deprivation, a relatively poor housing stock, poor health outcomes, and a transient population. In this challenging context, these findings are particularly illuminating and demonstrate the promise of this type of provision in supporting the housing needs of a vulnerable population with complex needs. However, we note that because of this context, the findings are not generalizable but may be relevant and transferable to other local contexts.

Given the lack of a substantial evidence base as discussed earlier, there are several possibilities for further research within the area of supported housing and the wider field concerning this population. For example, this study was undertaken with a small sample and was not able to follow residents’ individual journeys for any considerable period, and as such we are not able to take a longitudinal perspective on what happens to residents in the medium or long term. Further research that takes a longitudinal approach could provide deeper perspectives on the sustained impact of supported housing interventions. Additionally, further research conducted across multiple localities may be able to highlight if support needs or findings are consistent across multiple localities or whether there are any differences and, importantly, what the drivers are for any variations. While this study provides insight into residents experiences of supported housing, it does not explore causal links between these experiences and individual recovery outcomes. Nonetheless, understanding residents valued features of supported housing offers practical implications for public health and service design. For example, interventions can be tailored to prioritize staff training in lived experience-informed support, foster supportive community networks within housing, and ensure housing conditions promote autonomy.

It is important to acknowledge that this paper highlights one form of support for this population. Other types of housing interventions exist to support this population, such as Housing First which is gaining prominence in the United Kingdom and internationally (43–45). As we established earlier, there is a paucity of evidence for housing interventions with this population and particularly in the United Kingdom. Given this population often has different, often overlapping and multiple complex needs, another area for further research is to develop understanding on the efficacy of what housing interventions work best for groups where support needs are different. In other words, little to nothing is known about the comparative effectiveness of different housing interventions for this population.

## Conclusion

This study explored residents’ experiences in recovery and homelessness supported housing provision in the North-West of England. The findings highlight the role that meaningful relationships with staff, other residents, and the wider community have on residents’ sense of connection and belonging. The findings also highlight the role of the physical environment demonstrating how safe and secure spaces contribute to residents’ autonomy and align with wider psychological theories of ontological security. Overall, this study contributes to the wider literature by reinforcing the complex nature of supported housing and demonstrates the need for multifaceted support that considers both interpersonal and environmental needs.

## Data Availability

The datasets generated for this study consist of anonymized qualitative interview transcripts. Although all transcripts have been anonymized, they may still contain contextually sensitive information. For this reason, the data are not publicly available. Access to the anonymized transcripts can be requested from the corresponding author and will be considered on a case‑by‑case basis, subject to appropriate confidentiality safeguards and alignment with ethical approvals.
