# Supplementary material for: Going beyond: an exploration of residents’ experiences in recovery and homelessness supported housing provision in the United Kingdom
Source: Front Public Health. 2026 Apr 20;14:1783501. doi: 10.3389/fpubh.2026.1783501 (PMC13135937; doi:10.3389/fpubh.2026.1783501)
Supplement: Supplementary file 1 [file Data_Sheet_1.PDF]

Supported housing draft interview guide.

Key:

Numbered questions represent key questions

Alphabetical questions represent potential probing questions

Resident journey and environment

1. Could you walk us through your supported housing journey?

    a. Is this what you thought supported housing would be like? (probe around response)

2. Are you able to share a particular rule or policy that you find important or challenging to follow?

3. How would you describe your living environment?

    a. What do you like about it?

    b. What don't you like about it?

    c. Is there anything you would change or improve?

    d. Is there anything that would improve the environment for you and other residents?

Staff interactions

1. Do you get on with the people who work at your supported housing facility?

    a. Do you feel comfortable approaching them with questions or concerns?

    b. Are there any areas where you believe staff training or interactions could be improved?

2. Can you share any examples of staff support that have made a difference in your day-to-day life?

3. Do staff go out of their way to check up on you?

Safety, happy, cared for?

1. When it comes to safety, do you feel secure in your living environment? Can you tell me more about why or why not?

a. Is there a specific incident or moment when you felt particularly safe in this environment?

b. Are there any instances when you didn’t feel safe?

2. Relating to care, do you feel cared for in this space?

a. Do you mind sharing any experiences that have made you feel cared for and supported by the staff or other residents?

b. Are there any experiences or interactions that stand out to you?

3. Do you feel happy with this supported housing provider?

a. Are you able to share any specific factors that contribute to your happiness or unhappiness?

4. Are you able to share any positive experiences or challenges you have faced with others in supported housing?

a. Can you describe a memorable moment or experience that has had an impact on your experience in supported housing?

Daily activities

1. What does a typical day look like for you?

2. Are there any particular programs or activities offered here that you find enjoyable or beneficial?

a. Is there anything you would improve about these activities or programs?

3. Are there any additional activities or resources you would like to see offered?

Closing question:

1) If you had power to make changes to your supported housing, what would you change?

END
